# Mineralogical Characteristics and Luminescent Properties of Natural Fluorite with Three Different Colors

**DOI:** 10.3390/ma15061983

**Published:** 2022-03-08

**Authors:** Xiao Ge, Qingfeng Guo, Qianqian Wang, Tao Li, Libing Liao

**Affiliations:** 1 School of Gemology, China University of Geosciences, Beijing 100083, China; 2009200015@cugb.com (X.G.); 2109190009@cugb.edu.cn (Q.W.); 2009200013@cugb.edu.cn (T.L.); 2 Jewelry and Mineral Materials Laboratory of Experimental Teaching Demonstration Center, Beijing 100083, China; 3 Beijing Key Laboratory of Materials Utilization of Nonmetallic Minerals and Solid Wastes, National Laboratory of Mineral Materials, School of Materials Sciences and Technology, China University of Geosciences, Beijing 100083, China

**Keywords:** fluorite, REE, luminescent properties, temperature sensing

## Abstract

Fluorite is rich in mineral resources and its gorgeous colors and excellent luminescence characteristics have attracted the attention of many scholars. In this paper, the composition, structure, luminescent properties, and the potential application value of three fluorites with different colors and are systematically analyzed. The results show that REE and radioactive elements have effects on the structure, color, and luminescence of fluorite. Radioactive elements Th and U will aggravate the formation of crystal defects in fluorite. The green color is related to Ce^3+^ and Sm^2+^. Colloidal calcium and F^−^ center are responsible for the blue-purple color of fluorite. There are many luminescent centers, such as Eu, Pr, Dy, Tb, Er, and Sm, in fluorite. The blue fluorescence is mainly caused by 4*f*^7^-4*f*^6^5*d*^1^ of Eu^2+^. In addition, it is found that fluorite has certain temperature sensing properties in the temperature range of 303–343 K.

## 1. Introduction

Fluorite, also known as fluorspar, is named for its fluorescence under ultraviolet and cathode-ray irradiation. Its chemical formula is CaF_2_. Rare-earth elements (REEs), transition metal elements (Cr, Mn, Fe, Zn), and alkaline elements (Na, K) can often occupy Ca sites in the crystal structure of fluorite. Pure fluorite is colorless, but due to the existence of impurities and defects, fluorite presents different colors, such as purple, blue, green, yellow, and pink. A large number of scholars have had rich discussions on the chromogenic mechanism of fluorite. There are three main viewpoints, that is, the coloration of impurity elements [1,2,3,4,5], crystal defects [6,7,8,9], and organic matter [10,11]. REE^3+^, U^4+^, Th^4+^, and other elements widely exist in fluorite. Fluorite is an important reservoir of REE. From the perspective of crystal chemistry, the sites of Ca (1.06 Å) in the fluorite structure can be occupied by REE in the form of isomorphism. Under thermal and radiation conditions, the valence change, electron migration, and charge transfer of REE will lead to changes in the color of fluorite. For example, Sm^2+^, Dy^3+^, and Tm^2+^ are the reasons why fluorite is green. Red fluorite usually contains Gd^3+^, while yellow fluorite often contains Yb^3+^ [1,2,3,4]. On the other hand, fluorite has a variety of defects, including some defects that can lead to color. Fluorite and surrounding rocks often contain radioactive elements such as U and Th. Fluorite is easily irradiated by radioactive elements to form colloidal calcium. When colloidal calcium stays in lattice defects, fluorite produces a typical characteristic absorption band in the range of 560–580 nm. Under the influence of radiation, fluorite is also easy to form an F^−^ center [5,6,7]. In the UV-visible spectra, the absorption peaks of the F^−^ center are located at 375, 525, and 560 nm. The wide absorption band centered near 560 nm may be caused by the F^−^ center and colloidal calcium. With the enrichment of detection methods and in-depth research, the color of fluorite may be caused by many reasons [8,9,10]. In addition, organic matter sometimes exists in fluorite in the form of inclusions. Studies have shown that some dark fluorite will be mixed with organic matter. For example, asphaltene can exist in the form of solid inclusions in fluorite and make the color of fluorites darker. Fluorite in Huayuan lead-zinc mine in Hunan, China, is black because it contains solid hydrocarbons [4,11].

The existence of REE will not only change the color of fluorite but also lead to the fluorescence and phosphorescence of fluorite [12,13,14,15]. Studies have shown that in addition to the common blue fluorescence, some fluorites also show fluorescence of other colors, such as yellow, yellow-green, green, and red [16,17,18,19,20]. The discussion on the luminescence mechanism of fluorite has continued to be in-depth [21,22,23,24,25].

Under the excitation of ultraviolet light, most fluorite shows blue fluorescence with a strong emission peak near 425 nm caused by the 4*f*^7^-4*f*^6^5*d*^1^ transition of Eu^2+^ [26,27,28,29]. When the Eu^2+^ content is low, the luminescence center is taken by other rare-earth ions. Fluorites with yellow fluorescence have typical emission peaks at 483, 573, 653, and 750 nm. These peaks may be caused by the ^4^F_9/2_-^6^H_J_ transition in the 4*f*^9^ electronic configuration of Dy^3+^ (J = 15/2, 13/2, 11/2, and 9/2, respectively) [30,31,32,33]. Yellow-green fluorescence may be caused by Yb^2+^. At 364 and 264 nm, there are transitions from the ground state, ^1^S_0_, to the excited states, 4*f*^13^ (^2^F_7/2_), 5*d* (E_g_) and 4*f*^13^ (^2^F_5/2_), 5*d* (E_g_), in Yb^2+^. Samples that emit green fluorescence usually show emission peaks at 522, 539–549, and 666 nm, and the three emission peaks are respectively caused by ^2^H_11/2_-^4^I_15/2_, ^4^S_3/2_-^4^I_15/2_, and ^4^F_9/2_-^4^I_15/2_ transitions of Er^3+^. The red fluorescence of fluorite is caused by Er^3+^ and Sm^3+^ [34,35,36,37].

In addition to its beautiful appearance and charming luminous color, fluorite is also rich in mineral resources. Based on this, it is of great significance to deeply explore the production value of fluorite. However, so far, the research on the luminescent properties of natural fluorite and its potential applications in the field of luminescence has not been systematic. Therefore, this paper selects three samples of fluorite with different colors to discuss the composition, structure, and luminescence mechanisms of natural fluorite. The application of fluorite in the field of temperature sensing is further discussed. This manuscript is helpful to understand the optical properties of fluorite and broaden the application of fluorite. 

## 2. Materials and Methods

**Figure 1:**
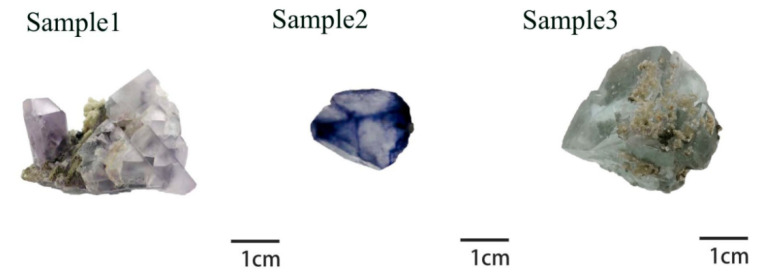
Photos of purple, blue, and light green fluorite samples.

Figure 1 shows three fluorite samples collected in Chenzhou City, Hunan Province, China. The colors of the three samples are purple (Sample 1), blue (Sample 2), and light green (Sample 3).

Electron probe tests were performed using an EPMA-1600 electron probe microanalyzer manufactured by Shimadzu Corporation, Japan. The test conditions were: acceleration voltage was 15 kV, the current was 10 nA, electron beam spot diameter was 5 μm, carbon spray on the sample surface, SPI standard sample, and ZAF calibration method for data processing.

Trace element detection was performed using Analytik Jena Plasma Quant MS laser ablation, inductively coupled plasma, mass spectrometry (LA-ICP-MS), Germany. The laser ablation system was the RESOlution 193 nm excimer laser. High-purity helium was used as the carrier gas in the experiment. The single point analysis time was 85 s, including 20 s for blank background collection, 45 s for continuous ablation collection, and 40 s for cleaning the sampling system. The sample acquisition data spot size was 100 μs.

The powder X-ray diffraction (XRD) instrument was the Dmax12kw powder diffractometer. Experimental conditions were: copper target, Kα Radiation source (λ = 0.15418 nm), the tube voltage was 40 kV, the tube current was 100 mA, the divergence gap and scattering gap on the goniometer was 1°, the scanning speed was 4°/min, and the sampling step was 0.02° (2 θ). The test range was 5–80°.

The infrared spectra test used the Tensor 27 Fourier infrared spectrometer, adopting the transmission method, and the experimental test conditions were as follows: test voltage was 220 V, the resolution was 4 cm^−1^, the scanning range was 4000–400 cm^−1^, and the scanning speed was 10 kHz.

The HR-Evolution Raman microscope produced by Horiba, Japan, was used. Experimental test conditions were: excitation light source was 532 nm, the grating was 600 (500 nm), the test range was 2000–100 cm^−1^, and the integration time was 3 s.

A UV-Vis spectrophotometer of the model UV-3600 produced by the Shimadzu factory in Japan was used. The experimental test method was the reflection method, the test range was 200–900 nm, the light source conversion wavelength was 300 nm, the grating conversion wavelength was 850 nm, and the sampling interval was 0.5 s.

The Hitachi F-4700 instrument was used to measure the optical absorption spectra, fluorescence emission (PL), and excitation (PLE) spectra of the samples. At the same time, a fluorescence spectrometer (FS 5, Edinburgh) was used to measure the attenuation curve of the samples, and a computer-controlled heating accessory was connected to the spectrometer to record the PL spectrum at 303–543 K.

## 3. Results and Discussion

### 3.1. EPMA

The Supplementary Table S1 shows the EPMA test results of fluorite. The results show that the fluorite samples were mainly composed of Ca and F elements. Trace elements in the samples included transition metal elements (Cr, Mn, Fe, Zn), alkaline earth elements (Na, K), and REE (Ce, Y). In the three samples, the average mass fractions of Ca were 51.318, 49.735, and 52.565 wt.%, which are close to the theoretical mass fraction of Ca, at 51.33 wt.%. The average mass fractions of F were, respectively, 48.571, 46.722, and 46.451 wt.%, which are lower than the theoretical value of 48.67 wt.%. This deviation is because the impurity ions in the fluorite lattice replaced F^−^ and Ca^2+^, such as REE^3+^ + F^−^→Ca^2+^, 2Ca^2+^→REE^3+^ + Na^+^, etc. 

### 3.2. LA-ICP-MS 

**Figure 2:**
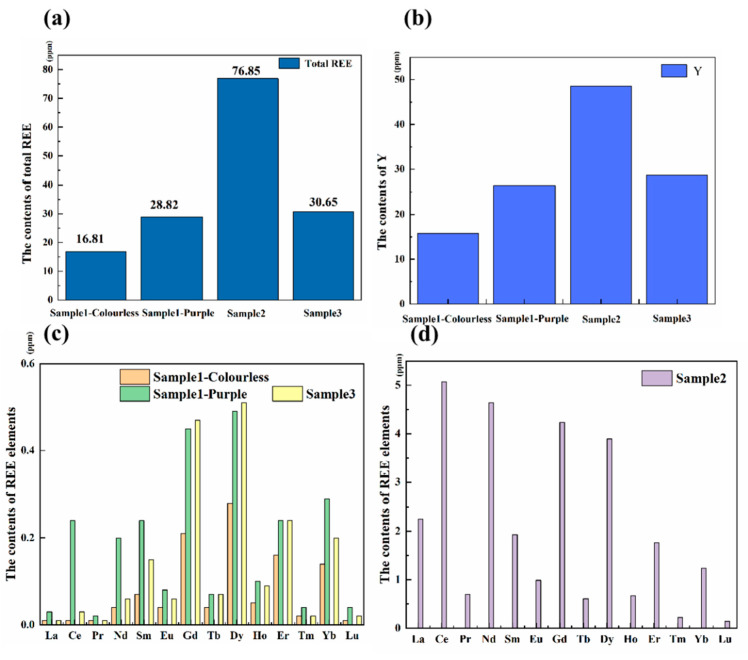
(**a**) The total content of REE in the samples. (**b**) The content of Y in the samples. (**c**) The content of each rare-earth element (except Y) in the sample 1 (colorless and purple parts) and sample 3. (**d**) The content of each rare-earth element (except Y) in the sample 2.

The Supplementary Table S2 and Figure 2 shows the LA-ICP-MS analysis results. Fluorite contains rich trace elements such as Na, Mn, Fe, Cr, Si, Y, La, Ce, Pr, etc. The content of rare-earth ions plays an important role in the color and luminescence of fluorites. Figure 2a shows the total amount of REE, Figure 2b shows the content of Y in the samples, Figure 2c shows the content of each REE in sample 1 (colorless and purple parts) and sample 3, and Figure 2d shows the content of each REE (except Y) in sample 2. The results showed that the content of REE in the deep-colored sample (sample 2) was higher than that in other samples. The distribution of rare-earth contents in the colorless part and the purple part of sample 1 is also different. Therefore, it can be seen that dark fluorite is often enriched in REE.

### 3.3. XRD

**Figure 3:**
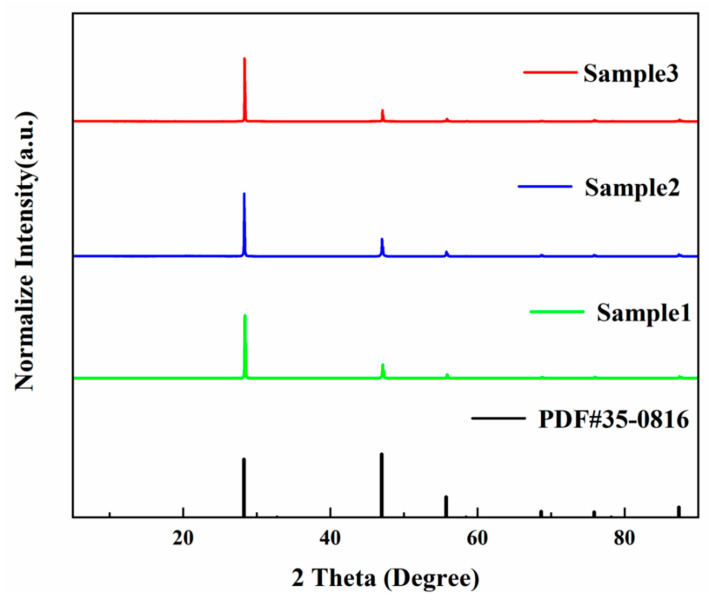
XRD patterns of the three samples and standard data of fluorite (JCPDS No. 35-0816).

Figure 3 shows the XRD patterns of the three samples. The standard data of fluorite (JCPDS No. 35-0816) are shown as a reference. We normalized the diffraction peak intensity of fluorite samples by using Origin 2018 software. The results show that all the XRD patterns of the three samples matched perfectly with that of the reference JCPDS file [4,20]. However, according to the Bragg diffraction formula (Formula (1)), the diffraction peak of the sample shifted slightly to the larger angle side, compared to the standard card:

(1) $$d_{hkl}=\frac{\lambda }{sin\theta_{hkl}}$$

It can be seen that the right shift of the diffraction peak is related to the decrease the of cell parameters. The unit cell parameters of the three samples were calculated by JADE software (Table 1), which shows that the cell parameters and cell volume of the three samples were slightly lower than that of the standard card (a = 5.4631 Å, V =163.0 Å^3^). This is because, during the growth of fluorite, a large number of small-radius impurity elements (transition metal elements such as iron and manganese and REEs) replace the original calcium element.

### 3.4. Infrared Spectra

**Figure 4:**
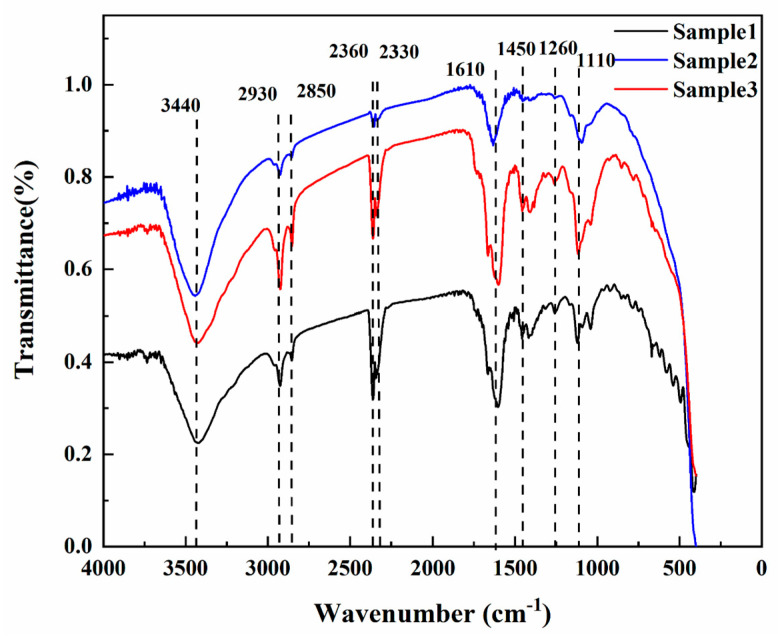
FTIR spectra of the three fluorite samples.

Figure 4 shows the infrared spectra of the three fluorite samples. The characteristic absorption peak of CaF_2_ near 1110 cm^−1^ exists in all the infrared spectra of the three samples, which is consistent with the characteristic peak of fluorite at 1080 cm^−1^ recorded in mineral spectroscopy [37]. The three samples all show a wide absorption band near 3440 cm^−1^ caused by OH stretching vibration, indicating the existence of constitutional water in fluorite. The same phenomenon also exists in fluorite from India and Mexico [38,39,40,41]. The absorption peaks around 2930 and 2850 cm^−1^ are caused by organic groups that may be due to the adsorption of oleic acid ester on the surface of fluorite [42]. The two absorption peaks at 2360 and 2330 cm^−1^ are caused by the asymmetric stretching vibration of CO_2_ [4,21]. The generation of 2360 and 2330 cm^−1^ may be related to the inclusion of CO_2_ in the fluorite or the CO_2_ in the air [43,44]. The absorption peaks at 1610, 1450, and 1260 cm^−1^ are caused by CO_3_^2−^ stretching vibration, and the free CO_3_^2−^ may be related to the CaCO_3_ contained in fluorite [45,46,47].

### 3.5. Raman Spectra

**Figure 5:**
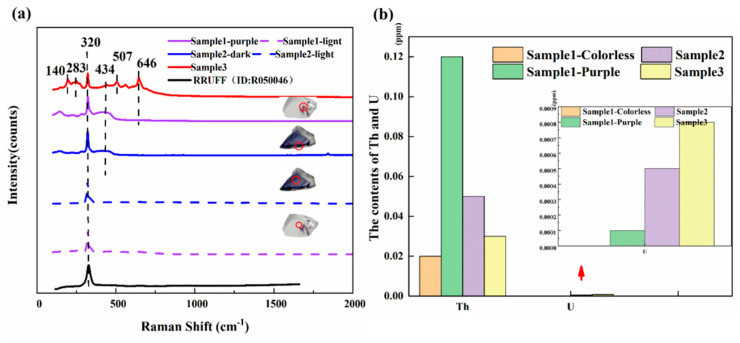
(**a**) Raman spectra of the three studied fluorite samples, and (**b**) the contents of Th and U in samples.

Figure 5a shows the Raman spectra of the three samples. The colorless part of sample 1 and the light part of sample 2 only showed a peak at 320 cm^−1^, which is consistent with the fluorite peak in the RRUFF database (ID: R050046). This peak originates from the fact that fluorite has only one T_2g_ Raman active vibrational peak [48,49,50,51]. In the deep-color part of these samples, peaks appeared at 140, 283, 434, 507, and 646 cm^−1^, which may be related to irradiation [50,51,52]. Before irradiation, only a single T_2g_ Raman active band was observed at 320 cm^−1^, and with the increase of irradiation fluence, the peak intensity of 140, 283, and 434 cm^−1^ in the samples also increased. Figure 5b shows the radioactivity determined by the LA-ICP-MS. The deep-color part also accumulated more Th and U elements. Therefore, it is speculated that the impurity peaks in the Raman spectra are related to the fluorite crystal defects caused by impurity ions (REE^3+^) or the radioactive elements Th and U.

### 3.6. UV-Vis Spectra

**Figure 6:**
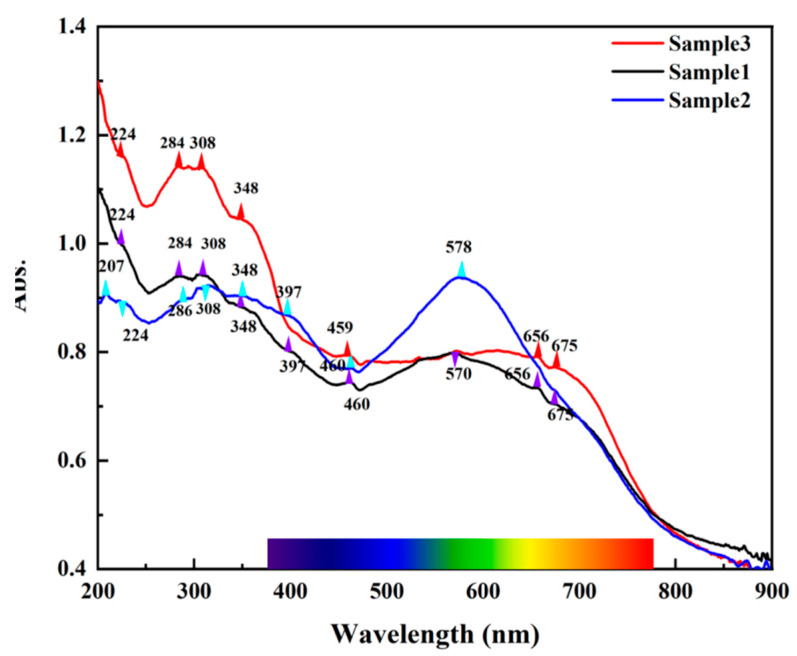
UV-Vis spectra of the three fluorite samples.

The presence of REE and other impurity elements will not only affect the crystal structure of fluorite but also make fluorite show different colors [1,2,3,4,5]. Figure 6 shows the UV-visible spectra of the three different colored samples. The purple and blue samples have a wide absorption band centered near 570 and 578 nm, respectively. The complexity of the spectra indicates that there are defects in the fluorite structure that may lead to the color of fluorite. With the deepening of research, the formation of each color seems to be due to more than one reason [6,7,8,9]. All samples have absorption peaks at 224, 284, 348, and 459 nm, but these absorption peaks may not affect the color of fluorite. These four absorption peaks are more likely to be the absorption peaks of a certain impurity ion crystal field or a certain charge transfer peak. The 224 nm absorption band is caused by the electrons trapped in the Ca^2+^ interstitial and the 348 nm absorption band is caused by the holes trapped in the Ca^2+^ vacancy. However, the Y element will strengthen the absorption peaks at 224, 348, and 400 nm [10,11]. The absorption peak at 460 nm may be related to the YO_2_ color center composed of Y^3+^ and O_2_^3−^ [11]. The wide absorption bands centered at 570 and 578 nm indicate the presence of colloidal calcium and F^−^ center, and the absorption peaks at 309, 645, and 675 nm may be related to Ce^3+^ and Sm^2+^ [1,2,3,4,5,6]. In the visible range, the three samples’ colors are mainly related to the purple and yellow parts of the absorption spectra. Therefore, it is speculated that the absorption peak at 570 nm is the reason for the blue-purple hue of fluorite samples, while the absorption peaks at 656 and 675 nm may be related to the yellow-green hue.

### 3.7. Photoluminescence Spectroscopy

**Figure 7:**
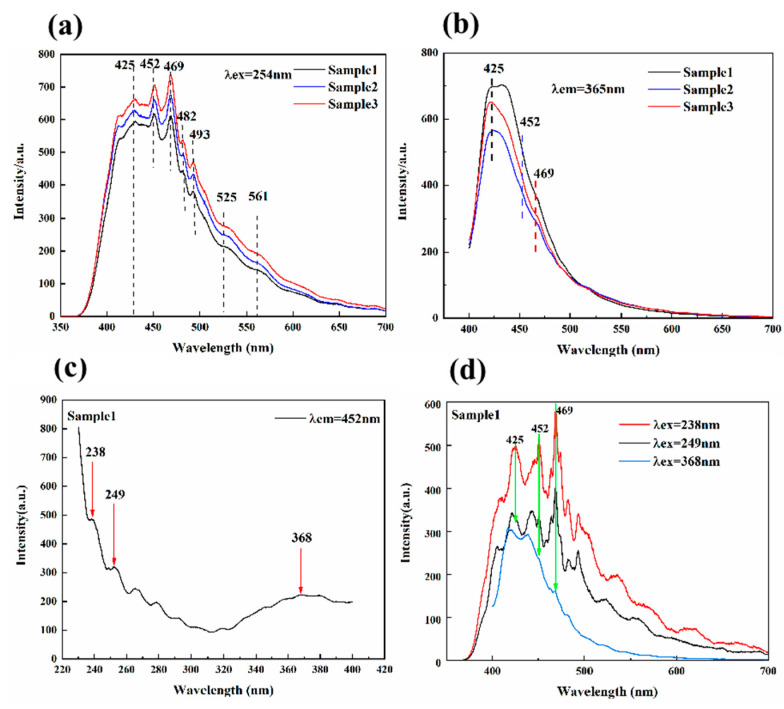
(**a**,**b**) The photoluminescence emission spectra of the samples (λ_ex_ = 254, 365 nm). (**c**) The excitation spectrum of sample 1 (λ_em_ = 452 nm). (**d**) The emission spectrum of sample 1 (λ_ex_ = 238, 249, 368 nm).

Figure 7a,b show the photoluminescence emission spectra of the samples (λ_ex_ = 254, 365 nm), Figure 7c shows the photoluminescence excitation spectrum of sample 1 (λ_em_ = 452 nm), and Figure 7d is the photoluminescence emission spectra of sample 1 (λ_ex_ = 238, 249, 368 nm). When λ_ex_ was 238 nm, the three main emission peaks at 425, 452, and 469 nm had the highest intensity. Therefore, λ_ex_ = 238 nm was used as the excitation wavelength of the heating experiment.

The strong emission peaks of the three samples were 425, 452, and 469 nm, accompanied by weak emission peaks at 482, 493, 525, and 561 nm. The complex emission peaks in fluorite were also caused by REE, which is rich in energy-level transitions. Table 2 summarizes the possible formation mechanisms of the emission peak. The broad emission centered at 425 nm was caused by the 4*f*^7^-4*f*^6^5*d*^1^ transition of Eu^2+^ [21,22,23,24,25]. The luminescence center at 452 nm may be caused by ^1^D_2_-^3^H_4_ of Tm^3+^ [26,27,28,29,30]. The emission peak at 469 nm was caused by ^3^H_4_-^3^P_1_ of Pr^3+^ [31,32]. The adjacent emission peaks at 482 and 493 nm may be due to ^1^G_4_-^3^H_6_ of Tm^3+^, ^5^D_4_-^3^F_6_ of Tb^3+^, ^4^I_15/2_-^4^F_7/2_ of Er^3+^, ^4^F_9/2_-^6^H_15/2_ of Dy^3+^, and ^3^H_4_-^3^P_0_ of Pr^3+^ [32,33]. Er^3+^, Dy^3+^, or Sm^3+^ may cause emission peaks at 525 and 562 nm [34,35,36].

**Figure 8:**
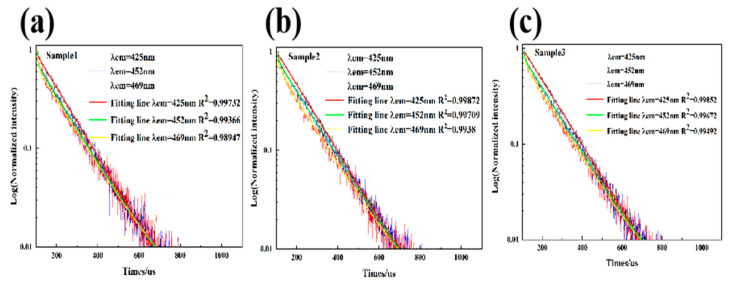
The photoluminescence decay curves of 425 (**a**), 452 (**b**), and 469 nm (**c**) emission peaks of the three samples.

Figure 8 shows the photoluminescence decay curves of the three samples. The decay curves can be fitted by a double exponential equation [53]:

I(t) = I_0_ + A_1_exp(−t/τ_1_) + A_2_exp(−t/τ_2_) (2)

Among them, I(t) and I_0_ are the luminous intensity and background intensity of time, t, respectively, A_1_ and A_2_ are emission intensity factors, and τ_1_ and τ_2_ are, respectively, the attenuation times of the exponential component [54]. The average lifetime is calculated by the following equation:

τ_ave_ = (A_1_τ_1_^2^ + A_2_τ_2_^2^)/(A_1_τ_1_ + A_2_τ_2_) (3)

Based on Equation (3), Table 3 shows the luminescence lifetimes of the three samples at 425, 452, and 469 nm (λ_ex_ = 238 nm). The luminescence lifetimes of the three emission peaks were 129 to 130, 8 to 123, and 9 to 43 ms, respectively. The luminescence lifetime of sample 2 at 452 nm was 8 ms, and the luminescence lifetime of sample 3 at 469 nm was 9 ms. The luminescence lifetime of Eu^2^ at 425 nm was consistent with previous studies, mostly 0.6–0.8 μs, and a few luminescence lifetimes can reach several ms. The prolonged luminescence lifetime may be caused by the energy migration of other long luminescence center ions in the samples. The emission peaks at 452 and 469 nm may be related to Tm^3+^ and Pr^3+^, respectively. The luminescence lifetime of Tm^3+^ is mainly 30–35 μs, and the luminescence lifetime of Pr^3+^ is mostly in the range of 5–10 μs [13,25]. The luminescence lifetimes of the three samples at 452 and 469 nm were different from previous studies. The reason may be that fluorite has multiple emission centers in the wavenumber range of 450–500 nm, such as Pr^3+^, Dy^3+^, Tb^3+^, Er^3+^, etc. The emission peaks of different luminescence centers shielded from each other, or energy transfer occurred, resulting in changes in the luminescence lifetime of the luminescence centers. The blue fluorescence of the samples was mainly caused by Eu^2+^, which has high luminous intensity and the longest luminous lifetime.

**Figure 9:**
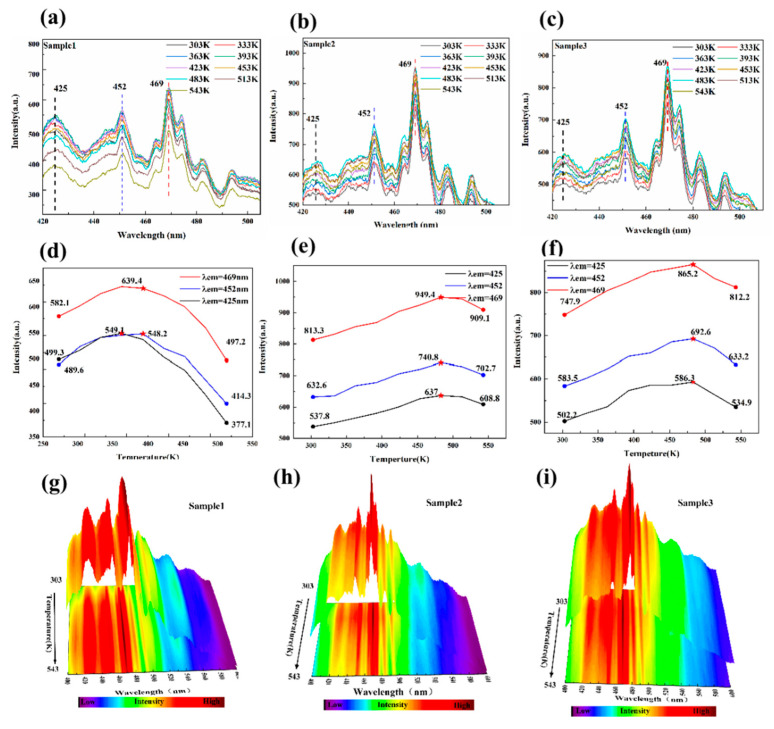
(**a**–**c**) The two-dimensional fluorescence spectra of fluorite of the three samples in the temperature range from 303 to 543 K. (**d**–**f**) The variation of luminescent intensity with the temperature at the three main emission peaks of 425, 452, and 469 nm (λ_ex_ = 238 nm). (**g**–**i**) The three-dimensional spectra of fluorite of the three samples in the temperature range from 303 to 543 K, respectively.

To obtain the effect of temperature on the luminescent properties of fluorite, the luminescence spectra of the three samples in the temperature range from 303 to 543 K were measured. Figure 9a–c show the two-dimensional fluorescence spectra of fluorite of the three samples in the temperature range from 303 to 543 K. Figure 9d–f show the variation of luminescent intensity with the temperature for the three main emission peaks of 425, 452, and 469 nm (λ_ex_ = 238 nm). Figure 9g–i display the three-dimensional spectra of fluorite of the three samples in the temperature range from 303 to 543 K, respectively. The results show that with the increase of temperature, the main luminescence positions for the emission peaks of fluorite had no significant changes. The intensity of the three strong emission peaks at 425, 452, and 469 nm first increased and then decreased. During the heating process (303–543 K), the luminous intensity of the three samples was almost higher than those at room temperature. Under the excitation of 238 nm, the higher the content of REE, the greater the luminous intensity of the main emission peaks. The luminous intensity of the main emission peaks of sample 2 was always higher than that of samples 1 and 3.

**Figure 10:**
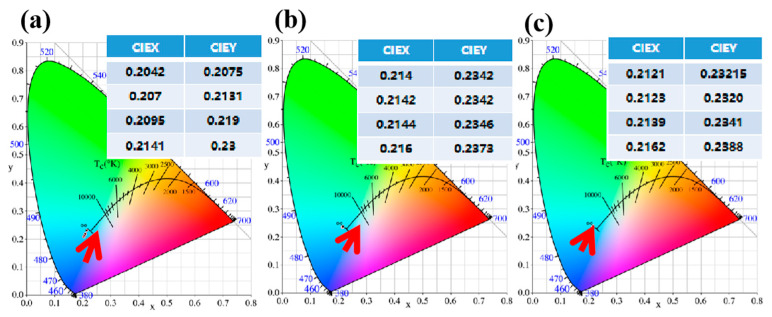
(**a**–**c**) The CIE chromaticity coordinates (x, y) of the three samples at 303, 393, 483, and 543 K.

Figure 10a–c reveal the emission colors and CIE chromaticity coordinates (x, y) of the three samples at 303, 393, 483, and 543 K. As the temperature continued to rise, the luminescent color of fluorite showed a red shift, but remained in the blue area. During the heating process, fluorite exhibited excellent luminescence stability. Mott formula was used to describe the thermal behavior of sample 3. The strong emission peaks at 469 and 493 nm were selected for discussion.

**Figure 11:**
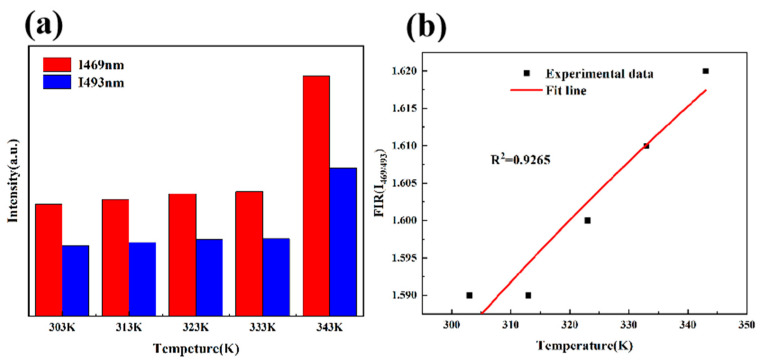
(**a**) The luminous intensity of the two emission peaks at 469 and 493 nm in the range of 303–343 K. (**b**) The fitting curve FIR (I_469_ nm/I_493_ nm) in the temperature range of 303–343 K.

Figure 11a shows the luminous intensity of the two emission peaks at 469 and 493 nm in the range of 303–343 K. Figure 11b reveals the fitted curve FIR (I_469 nm_/I_493 nm_) in the temperature range of 303–343 K. The parameters can be derived according to Equation (4):

(4) $$\mathrm{FIR}=\frac{I_{469\mathrm{nm}}}{I_{493\mathrm{nm}}}=A\;\exp\left(-\frac{\Delta E}{\mathrm{kT}}\right)$$

In Equation (4), the values of A and ΔE/KT are 1.87971 and 51.54436, respectively. The results show that the fluorite has a certain temperature sensing significance in this temperature range.

## 4. Conclusions

In summary, the composition, structure, and luminescence mechanisms of the purple, blue, and light green fluorite samples were studied. The results showed that the color of purple fluorite and blue fluorite was mainly caused by the broad absorption peak centered near 570 nm. The formation of this absorption peak is related to colloidal calcium and the F^−^ center, and the yellow-green hue of fluorite is related to trace amounts of Ce and Sm. During the heating process of 303–543 K, the luminous intensity and luminous color were relatively stable. In addition, this study showed that natural fluorite has a certain temperature sensing significance in the range of 303–343 K.

## Figures and Tables

**Table 1:** The cell parameters, cell volume, and fitting degree of the three samples.

Sample	a, b, c (Å)	Cell Volume (Å^3^)	Fitting Profiles (R)
1	5.45278	162.13	7.52
2	5.46082	162.93	8.52
3	5.45843	162.63	11.49
PDF#35-0816	5.46305	163.0	

**Table 2:** Emission lines and electronic transitions of natural fluorite.

Possible REE Ions	Emission Lines (nm)	Electronic Transitions	Reference
Eu^2+^	425	E_g_-^8^S_7/2_	[21,22,23,24,25]
Tm^3+^	452	^1^D_2_-^3^H_4_	[26,27,28,29,30]
Er^3+^		^4^I_15/2_-^4^F_5/2_	
Pr^3+^	469	^3^H_4_-^3^P_1_	[31,32]
Tm^3+^	482 and 493	^1^G_4_-^3^H_6_	[32,33]
Tb^3+^		^5^D_4_-^7^F_6_	
Er^3+^		^4^I_15/2_-^4^G_7/2_	
Dy^3+^		^4^F_9/2_-^6^H_15/2_	
Pr^3+^		^3^H_4_-^3^P_0_	
Er^3+^	525	^2^H_11/2_-^4^I_15/2_	[34,35]
Er^3+^	561	^4^S_3/2_-^4^I_15/2_	[35,36]
Sm^3+^		^4^G_5/2_-^6^H_5/2_	
Dy^3+^		^4^F_9_-^6^H_13/2_	

**Table 3:** The luminescence lifetime of the three samples for 425, 452, and 469 nm (λex = 238 nm).

Sample Name	λ_em_ = 425 nm	λ_em_ = 452 nm	λ_em_ = 469 nm
Sample 1	0.129 ms	0.125 ms	0.009 ms
Sample 2	0.129 ms	0.008 ms	0.043 ms
Sample 3	0.130 ms	0.119 ms	0.040 ms

## Data Availability

The study does not report any data.

## Supplementary Materials

The following supporting information can be downloaded at: https://www.mdpi.com/article/10.3390/ma15061983/s1; Table S1: The EPMA results of fluorite; Table S2: The LA-ICP-MS results of fluorites.
